# Using explainable machine learning and fitbit data to investigate predictors of adolescent obesity

**DOI:** 10.1038/s41598-024-60811-2

**Published:** 2024-05-31

**Authors:** Orsolya Kiss, Fiona C. Baker, Robert Palovics, Erin E. Dooley, Kelley Pettee Gabriel, Jason M. Nagata

**Affiliations:** 1 Center for Health Sciences, SRI International, 333 Ravenswood Ave, Menlo Park, CA 94025 USA; 2 Department of Pediatrics, University of California, San Francisco, San Francisco, CA USA; 3 Department of Neurology and Neurological Sciences, Stanford University School of Medicine, Stanford, CA USA; 4 Department of Epidemiology, University of Alabama at Birmingham, 1665 University Boulevard, Birmingham, AL 35233 USA; 5 School of Physiology, University of the Witwatersrand, Parktown, Johannesburg, South Africa

**Keywords:** Paediatrics, Weight management, Circadian rhythms and sleep, Computational neuroscience

## Abstract

Sociodemographic and lifestyle factors (sleep, physical activity, and sedentary behavior) may predict obesity risk in early adolescence; a critical period during the life course. Analyzing data from 2971 participants (M = 11.94, SD = 0.64 years) wearing Fitbit Charge HR 2 devices in the Adolescent Brain Cognitive Development (ABCD) Study, glass box machine learning models identified obesity predictors from Fitbit-derived measures of sleep, cardiovascular fitness, and sociodemographic status. Key predictors of obesity include identifying as Non-White race, low household income, later bedtime, short sleep duration, variable sleep timing, low daily step counts, and high heart rates (AUC_Mean_ = 0.726). Findings highlight the importance of inadequate sleep, physical inactivity, and socioeconomic disparities, for obesity risk. Results also show the clinical applicability of wearables for continuous monitoring of sleep and cardiovascular fitness in adolescents. Identifying the tipping points in the predictors of obesity risk can inform interventions and treatment strategies to reduce obesity rates in adolescents.

## Introduction

Obesity is a significant public health problem for adolescents in the United States^1^ with differences noted by race/ethnicity and socioeconomic status^1^. The prevalence of obesity in adolescence, which frequently persists into adulthood^2,3^ has significantly increased in past decades. One in six adolescents is classified as having obesity and one-third are at risk for obesity^4^ Early adolescence (10–14 years) is a particularly high-risk period for the development of obesity^5^ and carries a high risk for adult obesity^2,3^ and comorbid conditions, including earlier age onset^6^. Adolescent obesity also has immediate consequences, including advanced pubertal onset^7^, menstrual irregularities in girls^8^, and cardiovascular risk factors, including prediabetes, type 2 diabetes, high cholesterol levels, hypertension, and metabolic syndrome^9^. Additionally, adolescents with obesity may experience weight stigma and are at risk for psychological problems such as depression^10^, anxiety^11^, poor self-esteem and body image, poor peer relationships, and eating disorders^12^. The rarity of transitions from obesity to normal weight during adolescence^13^ shows that without targeted efforts, obesity becomes a persistent condition with long-term detrimental consequences.

Wearable devices, such as fitness trackers, smartwatches, and other health monitoring devices, have gained popularity among various age groups, including adolescents^14^. Wearable devices can record sleep, physical activity, and physiological measures (e.g., heart rate) that may be associated with obesity. Recently, several studies documented the use and efficacy of wearable devices to prevent and treat obesity in adults^15–17^.

Sleep problems are now recognized as being associated with obesity risk in adolescence^18^. Sleep is crucial for energy restorative processes and metabolism, mediating glucose metabolism^19^, influencing food intake^20,21^, and appetite regulation. Importantly, sleep changes considerably in adolescence, driven by biological and environmental/social factors leading to later bedtimes while still needing to get up early for school^22^. Consequently, youth are at risk for poor/insufficient sleep, especially those with high socioeconomic disparities^23,24^ and there is a growing number of adolescents in the US who are not getting sufficient, good quality sleep. With the rise of wearables, there's an added advantage in continuously detecting and tracking measurable behaviors, such as sleep and movement patterns and indices of cardiovascular (CV) fitness, which could be pivotal risk factors for obesity in adolescents. These devices provide an objective assessment of physical activity and sleep that addresses the limitations associated with self-report methods, including records, recalls, or questionnaires.

Physical inactivity in adolescents is another risk factor for obesity^25^, and overweight and obese youth are more sedentary and less physically active than their peers with healthy weight^26^. Increasing physical activity is considered an important strategy to increase physical activity related energy expenditure^27^, making exercise one of the most common strategies in the treatment and prevention of obesity^28^. In association with physical inactivity and obesity, heart rate variability tends to be lower and cardiovascular function is altered^29^ since the heart has to work harder to pump blood to the excess fat tissue, which requires more oxygen and nutrients^30^. As a result, the heart rate increases to compensate for this extra workload^31^.

Here we leverage the Adolescent Brain Cognitive Development (ABCD) study®, which collects data from a diverse adolescent population across the United States, to apply a data-driven approach relying on data collected with commercially available wearable Fitbit devices to identify the most important measurable predictors of obesity and investigate the non-linear associations of these predictors with obesity. This analysis allows us to pinpoint the tipping points in the predictors where the risk of obesity begins to escalate, shedding light on the complex interplay between lifestyle factors and health outcomes. In addition to sleep, the number of steps, cardiovascular fitness measures, and their day-to-day variability, we consider sociodemographic measures, shown to be relevant in the prediction of obesity^32^. We hypothesize that models trained primarily on wearable-derived measures will accurately predict adolescent obesity.

## Methods

### Participants

Data were obtained from the US-based, multi-site ABCD Study® (data version 5.0 released in June 2023), (21 research sites from 17 states—https://abcdstudy.org/study-sites/) of ~ 11,800 children aged 9–10 years at baseline^33^. Full details on the ABCD Study protocols, design, and recruitment procedures may be found elsewhere^33,34^. We included data from *2*,971 adolescents from the Year 2 assessment who had anthropometric data, and who provided data from the second and third weeks of the Fitbit protocol. As part of the preprocessing, we excluded participants who did not have at least two weekend and two weekday sleep data and participants with incorrect values (e.g., negative time in bed values). Written informed consent and assent were obtained from a parent/guardian and the adolescent, respectively. Procedures were approved by a centralized institutional review board (University of California, San Diego). All procedures complied with the Declaration of Helsinki. Table 1 provides a detailed breakdown of the demographics for our analytical sample. When compared to the ABCD sample of participants who were excluded due to insufficient data, the analytical sample consisted of slightly younger participants, with an average age of 11.94 years (SD = 0.64) as opposed to 12.02 years (SD = 0.66). The sex distribution was also marginally different, with females making up 49% of the analytical sample versus 47% of the excluded group. The analytical sample had a higher representation of White individuals (62.5% vs. 48.4% in the excluded group) and Asians (7.1% vs. 5.5% in the excluded group), while it had fewer Latinos (16.4% vs. 17.3%) and significantly fewer Black individuals (9.5% vs. 23.6%). Socioeconomically, both the analytical sample and the excluded group reported similar household incomes below $75,000, at 31.3% and 31.0% respectively. As for parental education, only 8% of the analytical sample had parents with a high school education or less, compared to 12.9% in the excluded group.

**Table 1:** Demographics collected at baseline for the subset of ABCD Study participants included in the current analysis (Fitbit sub-sample).

Variable	ABCD Fitbit sub-sample N = 2971
Age (years) mean (range)	11.94 (10–14)
Sex	
Female	1470 (49.4%)
Male	1501 (50.5%)
Race/ethnicity	
Asian	212 (7.1%)
Black	287 (9.6%)
Latino	488 (16.4%)
Native American	91 (3.0%)
Other	23 (0.7%)
White	1859 (62.5%)
Unknown/not reported	11 (0.03%)
Household income	
Below $75,000 household income	930 (31.3%)
$75,000 or above household income	1857 (62.5%)
Unknown/not reported	184 (6.1%)
Parental education	
< High school diploma	240 (8.0%)
College or above	2726 (91.4%)
Unknown/not reported	5 (< 0.1%)

### Input measures included in the models

#### Demographics

We included the following caregiver reported sociodemographic information from the baseline year, collected between 2016 and 2018: Biological sex (female/male), sex and gender minority status (yes/no), race/ethnicity (White, Latino, Black, Asian, Native American, other race), number of siblings, household income (high income/low income as defined based on the household income, which approximated the median US household income), highest educational attainment of parents/caregiver (college or above/high school or less), immigration status (“anyone in your child's family born outside of the United States”—yes/no), marital status of the caregiver (married/ not married, where married and living with a partner were combined; widowed, divorced, separated, and never married were categorized as not married/not living with a partner), access to food, telephone service, gas—electric service (e.g., “needed food but couldn't afford to buy it or couldn't afford to go out to get it”—yes/no), housing, eviction (e.g., “didn't pay the full amount of the rent or mortgage because you could not afford it”—yes/no), heath care—doctor (“needed to see a doctor or go to the hospital but didn't go because you could not afford it”—yes/no) and number of people living at the same address. Age collected in months at the Year 2 assessment was converted to years.

##### Medical history

As part of the Year 2 assessment, caregivers reported their child’s recent medical visits and conditions. We included items about hospitalization, emergency room visits, medical visits, and medical conditions (sickle cell anemia, problems with the heart, kidney disease, diabetes, cerebral palsy, asthma).

##### Fitbit measures

At the Year 2 assessment (November 2018 and November 2020), ABCD Study participants were invited to wear a Fitbit Charge HR 2 (Fitbit Inc., San Francisco, CA) on their wrist for up to 21 days^35^ (for more detailed description see Supplementary Section 1). Fitbit has been validated against research-grade, gold-standard devices in children and adolescents^36^. Fitbit Charge HR 2 devices use proprietary algorithms to continuously measure biobehavioral features at up to a one-second sampling rate^35^ with a photoplethysmography and accelerometer. We utilized the daily aggregated physical activity and sleep measures, as it was released by the ABCD Data Management team (see Supplementary Section 1 for more information). Within each adolescent's 21-day study protocol, we excluded the first week (7 days) of data from our analysis of weekday variability in sleep and physical activity measures. This exclusion considered the influence of self-monitoring^37^ or the awareness of being observed^38^, which are known to alter behavior in response to being monitored. Our decision was based on our observation that during the first week, participants tended to have higher levels of physical activity compared to the subsequent weeks (Fig. S3, and Supplementary Section 2). We hypothesized that this may have been due to factors such as excitement or novelty associated with wearing the Fitbit device, as well as an increased awareness of being observed, which often leads individuals to modify their behavior in the short term. By focusing on data from the second and third weeks, we aimed to capture more habitual and stable patterns of behavior. This approach improves the accuracy of our estimates of weekday variability in sleep and cardiovascular fitness and physical activity measures, offering insights into adolescents' habitual behaviors rather than their temporary adjustments to new circumstances. In doing so, we mitigate the potential distortions in data caused by the initial reaction to the novelty of the device and the consciousness of being part of a study, ensuring that our analysis reflects participants' regular patterns of physical activity^39,40^ and sleep^41,42^, that is relevant in the prediction of obesity, and has less temporal variations. From the remaining Fitbit data, we included summary scores of sleep, activity, and cardiovascular fitness measures (mean) and calculated variability in all daily measures using data from the second and third weeks (standard deviation, difference between the minimum and maximum value for weekdays and weekends, and mean square successive difference^43^ on weekdays).

*Sleep* Variables and variability measures included sleep timing (bedtime, wake-up time), sleep duration, sleep onset latency, sleep inertia (time taken to get out of bed) on weekdays and weekend days separately, and mid-sleep timing. In our study, mid-sleep is defined as the midpoint of the sleep cycle, calculated using the formula: Mid-Sleep = Sleep Onset Time + (Sleep Duration / 2), where Sleep Onset Time is the time at which the subject falls asleep, and Sleep Duration is the total time spent asleep, thereby providing a quantitative measure to assess individual chronotypes and natural sleep patterns. In addition, we calculated the sleep efficiency (the ratio of the total time spent asleep to the total amount of time spent in bed intended for sleep) and the relative and absolute social jetlag.

*Physical activity—steps per day* Prior research has demonstrated that Fitbit devices offer an accurate and reliable estimation of daily step count as a measure of accumulated physical activity in adolescents over time^35,36^. All days (weekdays and weekends separately) with a minimum of 1000 steps per day (and > 600 min of wear time) were included, in line with prior studies^44,45^.

*Cardiovascular fitness measures (Fitbit)* Data included sleeping heart rate (HR) and daytime resting state HR, averaged across the second and third week, as well as day-to-day variability in these measures.

##### COVID-19

Given that the COVID-19 pandemic occurred during some of the Fitbit data collection period, we created a COVID data collection variable (yes/no) based on dates of wear, defining March 13, 2020, as the onset of the COVID-19 pandemic in the U.S.

#### Outcome measure—obesity

At the Year 2 Assessment, weight (Health-o-meter 844KL High-Capacity Digital Bathroom Scale; Jarden Corporation; Rye, NY) and height (Carpenter’s square, steel tape measure) were assessed by the interviewer at each site. Body mass index (BMI) was computed using the standard formula, weight (kilograms) divided by height (meters) squared (BMI = weight/height^2^). BMI was converted into sex- and age-specific percentiles in accordance with the Centers for Disease Control and Prevention (CDC) growth curves and definitions. Individuals were classified as having obesity if their BMI was ≥ 95th percentile^46^.

### Training and evaluation of classification models

During the feature selection process, we removed features having missing records above 98% and dropped quasi-constant (> 99%) features from the dataset. Even though Explainable Boosting Machine (EBM) algorithms, which were employed in our study, inherently possess a robustness to multicollinearity, we took steps to reduce collinearity among variables with the aim of simplifying the model and enhancing its interpretability. To identify redundant features and reduce collinearity among variables, we calculated the Spearman’s rho (ρ) correlation matrix and identified pairs of variables with large correlation values (|ρ*|* > 0.75). We compared these features with the outcome variable and kept the feature that showed a higher correlation with the outcome in the model. Missing items for predictor variables were replaced using k-Nearest Neighbors^47^ (implemented in the Python package sklearn.impute.KNNImputer with uniform weights). Figures were generated using the seaborn (Version 0.11) Python (Version 3.8.5) package^48^.

Model performance depends on the selection of their hyper-parameters (e.g., learning rate). To find the best performing hyper-parameter configuration, we performed a grid search by evaluating all possible configurations in a predefined range.

**Figure 1:**
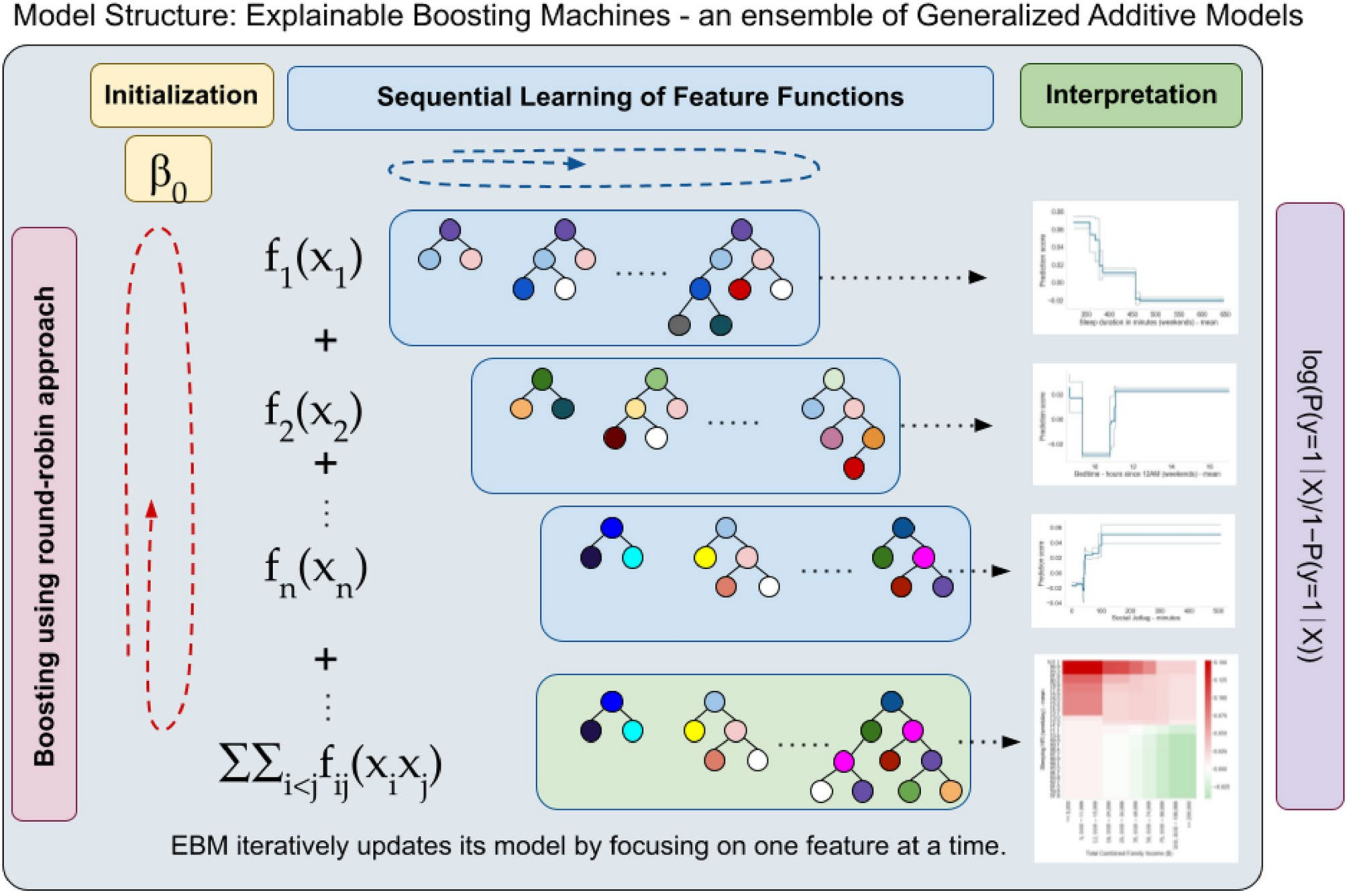
This figure illustrates the iterative process of building an Explainable Boosting Machine (EBM) for binary classification. Starting with a basic model, the EBM sequentially updates its predictions by cycling through each feature to learn its unique contribution via gradient boosting. This round-robin approach ensures gradual refinement, where each feature's effect is independently modeled and then aggregated to form the final predictive model. Interactions between pairs of features are also explored and integrated, enhancing the model's accuracy. The process emphasizes continuous learning from residuals, leading to a highly interpretable and precise predictive model that offers clear insights into the influence of individual features and their interactions on the outcome. P is the probability of the target variable belonging to the positive class (obesity), β0 is the intercept; fi (xi) represents the smooth function for the i-th feature; xi is the i-th feature; n is the total number of features^82^. Figures are just for illustration, generated using the seaborn (Version 0.11, URL: https://seaborn.pydata.org/generated/seaborn.heatmap.html) Python (Version 3.8.5) package^48^.

As our primary method, we employed the Explainable Boosting Machine (EBM)^49^ (implemented in the Python package InterpretML, 0.5.1), a non-linear machine learning model (Fig. 1) that can incorporate interactions among features. This ability is particularly useful when existing literature indicates potential interactions among the predictors, allowing us to construct a more informed and context-aware models. To identify the most important two-way interactions in the data, we included the number of interactions into the parameter search. For model evaluation, we used area under the receiver operating characteristic curve (AUC) metrics and accuracy (ACC). The AUC is a widely recommended measure for assessing models on unbalanced datasets due to its insensitivity to class distribution^50–52^.

To assess the performance of the EBM model in comparison to both linear and nonlinear models, we separately trained Regularized Logistic Regression with L1 (Lasso) regularization (referred to as LassoReg) and Gradient Boosted Trees (GBT). A comprehensive search across several potential parameters was conducted to identify the best-fitting model for targeting obesity.

For model comparison, we used a 2 × 5 cross-validation technique. We fitted the final model on the entire dataset using the implementation in scikit-learn^53^ (version 0.18.2) and Python 3.6.8.

### Feature importance in final models

We analyzed the global feature importance values of the fine-tuned EBM model that we trained on the entire dataset (lr = 0.005, max_round = 10, max_bin = 16, number of interactions = 5). We ranked the features according to their importance (absolute value of the coefficients) to interpret the best-performing models. For the sake of interpretability, we categorized the top features into eight domains: demographics (e.g., age, sex); cardiovascular health (e.g., HR); physical activity (e.g., step counts); medical history (e.g., during the past year seeing the doctor other than for regular checkups); and sleep (e.g., sleep duration).

### Implications and contribution

Using Explainable Boosting Machine algorithms, this study underscores the efficacy of wearable devices in predicting early adolescent obesity through sleep, physical activity, and socioeconomic factors. The wearables' convenience for health monitoring offers potential for early obesity intervention and promoting healthier behaviors, especially among socioeconomically disadvantaged adolescents.

## Results

In our analysis, the Explainable Boosting Machine algorithm illuminated the intricate relationships between the predictors and adolescent obesity, including key interactions among sociodemographic factors, sleep, physical activity, and cardiovascular fitness highlighted in Fig. 3. Figures 5 and Fig. S2 underscore the non-linear nature of these associations, revealing specific values and patterns that contribute to varying degrees of risk. This granularity in interpretation, made possible by the EBM's adeptness at handling non-linear relationships, offers a nuanced understanding of the intertwined roles of sociodemographic factors, sleep, physical activity and cardiovascular fitness in the context of adolescent obesity.

**Figure 2:**
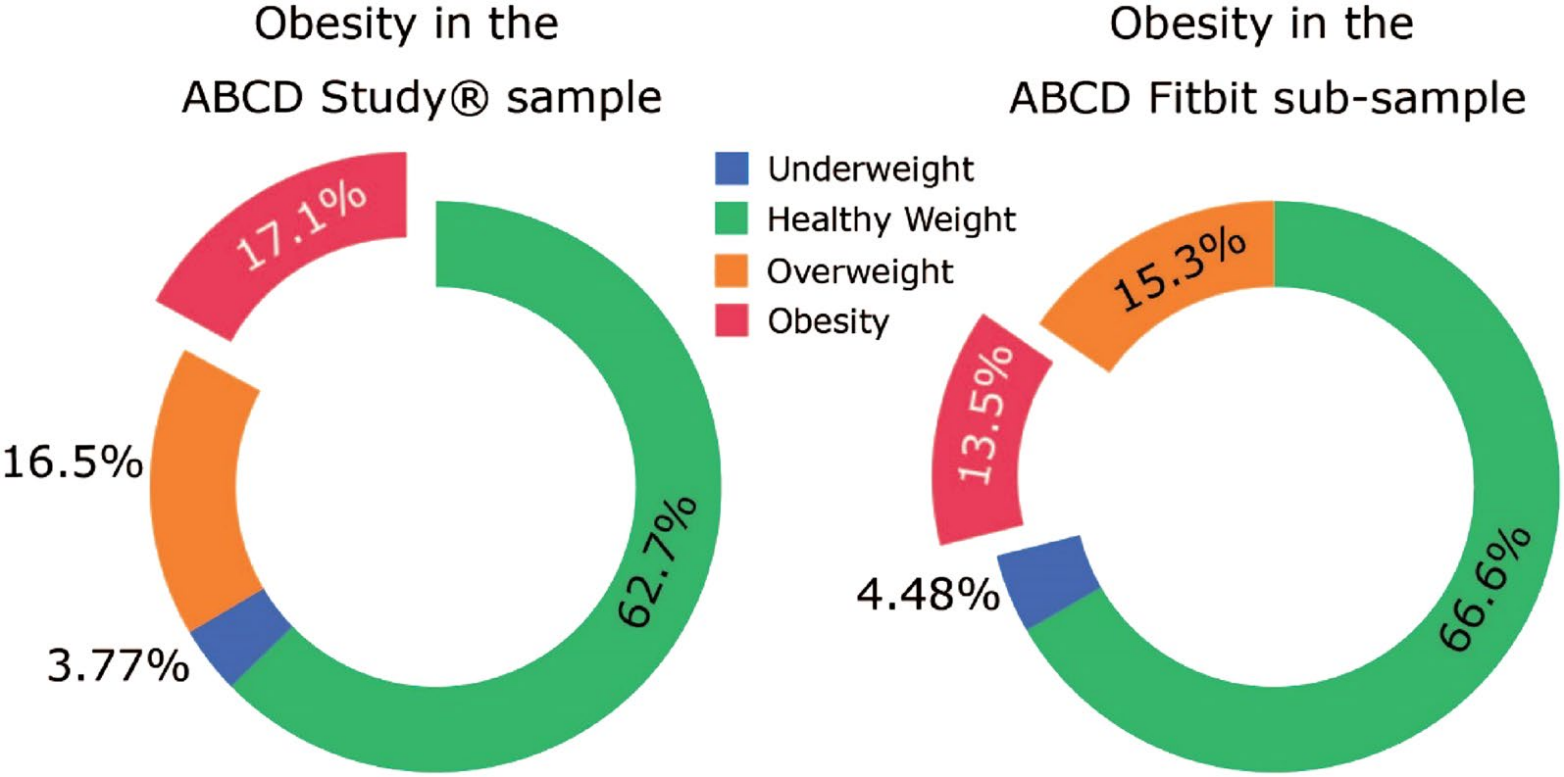
Body mass index distribution for the complete ABCD Study sample at the Year 2 assessment (N = 7552) and for the sub-sample of participants in the analytical sample with Fitbit and weight data (n = 2971) based on the CDC guidelines for BMI percentiles (Underweight: less than the 5th percentile; Healthy Weight: 5th percentile to less than the 85th percentile; Overweight: 85th percentile to less than the 95th percentile; Obesity: 95th percentile or greater).

The proportion of participants classified as having obesity in the Fitbit sub-sample analyzed here was lower (13.5%) than in the whole ABCD cohort at the Year 2 assessment (17.1%, Fig. 2). Results of the cross-validation indicated acceptable prediction performance for all our models; however, the EBM performed well both on the cross validation (mean AUC ≥ 0.726) and in the fine-tuned model (AUC ≥ 0.737) (Table 2).

**Table 2:** Results of the cross-validation and the hyperparameters for the final fine-tuned models predicting obesity in the ABCD sub-sample of adolescents with Fitbit and BMI data (N = 2971).

	Evaluation	LassoReg (Lr = 0.08)	Gradient boosted Tree (n_tree = 60, lr = 0.1, depth = 2)	Explainable boosting machine (max_round = 10, Lr = 0.005, max_bin = 16, interaction = 5)
2 × 5 fold cross validation mean performance	AUC	0.735	0.708	0.726
Best performing model	AUC	0.684	0.664	0.737
	ACC	0.86	0.65	0.86

### Feature importance in the classification model

**Figure 3:**
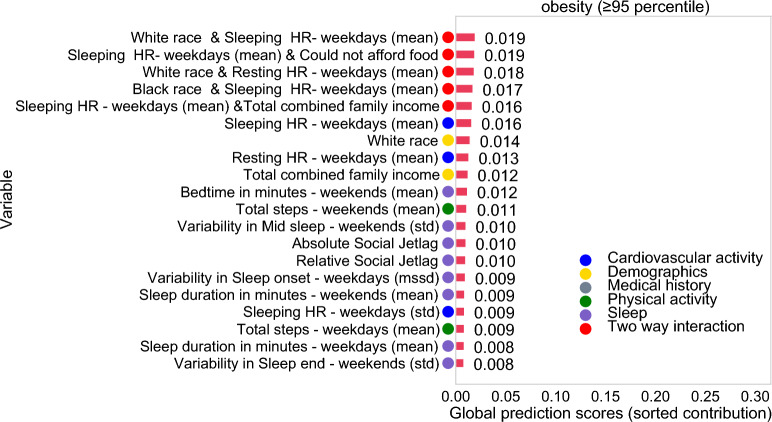
Mean absolute contribution score of each term (feature or interaction) in the best performing EBM model trained to predict obesity in young adolescents (n = 2971). The top 20 features are sorted by their contribution (*C*_*i*_), where *C*_*i*_ represents the contribution score for the *i-*th feature to the prediction. These scores are expressed in terms of log odds, derived from a combination of univariate models for each feature, considering their individual contributions as well as interactions with other features.

**Figure 4:**
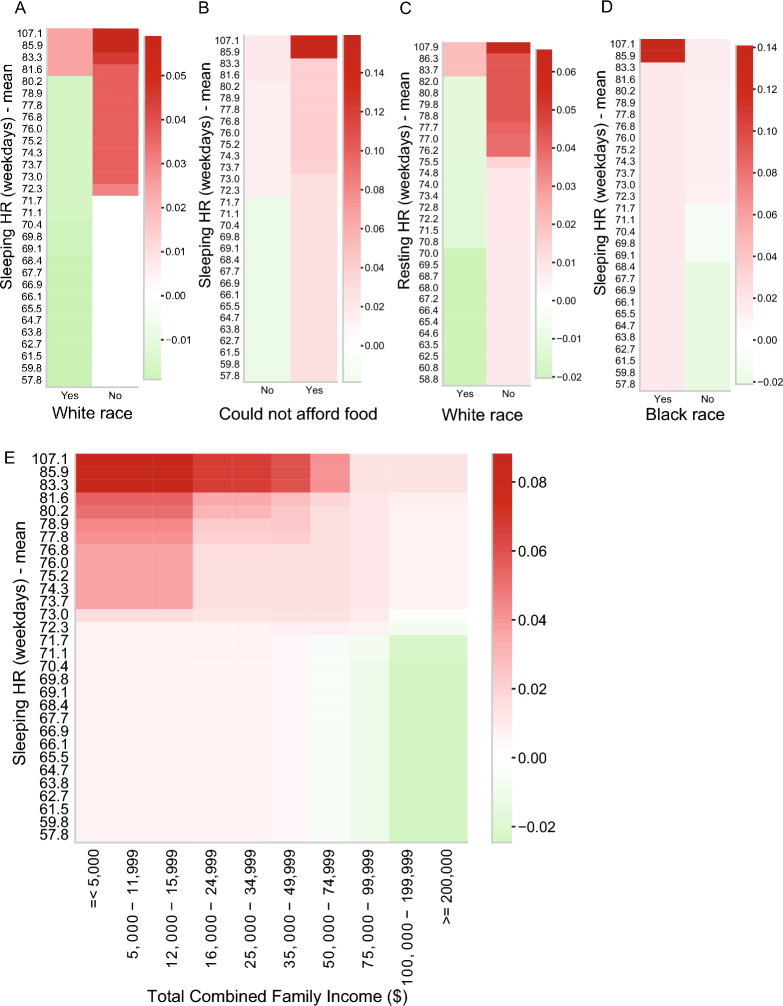
Interaction terms in the EBM model: two-way interaction between the Sleeping heart rate and White race (**A**), Sleeping heart rate and food access (**B**), White race and Resting heart rate (**C**), Black race and Sleeping heart rate (**D**) and the two-way interaction between the Sleeping heart rate and Household income (**E**) in the prediction of obesity. The color of the heatmap represents the direction of the effect (red: values associated with higher prediction score and increased risk, green: values associated with decreased risk). Figures were generated using the seaborn (Version 0.11, URL: https://seaborn.pydata.org/generated/seaborn.heatmap.html) Python (Version 3.8.5) package^48^.

The final EBM model included 69 features. Figure 3 shows the top 20 features by mean absolute contribution score (*C*). The model identified five important interactions influencing obesity risk. The interaction between White race and overnight heart rate emerged as the top-ranked contribution score (*C* = 0.019) within the model. This interaction reveals an elevated risk of obesity among individuals who identified as any race other than White who also have a sleeping HR greater than 72 BPM, and a corresponding increase in obesity risk among White individuals associated with a heart rate of 80 BPM or higher (Fig. 4A). Further interactions included White race with Resting HR (*C* = 0.018) and Black race with Sleeping HR (*C* = 0.017), both highlighting race-specific variations in heart rate associated with obesity risk (Fig. 4C-D). Another key interaction was between sleeping heart rate and economic hardship, measured by difficulties in affording food (*C* = 0.019). Participants facing food access issues with higher sleeping heart rates were at greater risk for obesity (Fig. 4B). This finding aligns with another interaction between Sleeping HR and Total Combined Family Income (*C* = 0.016), revealing nuanced risk profiles. Specifically, participants from families with incomes ranging between $5,000 or lower and $16,000 who also had elevated sleeping HR were found to be at the highest risk for obesity. Conversely, having a family income above $100,000 and a lower sleeping HR was associated with a decreased risk of obesity (Fig. 4E).

**Figure 5:**
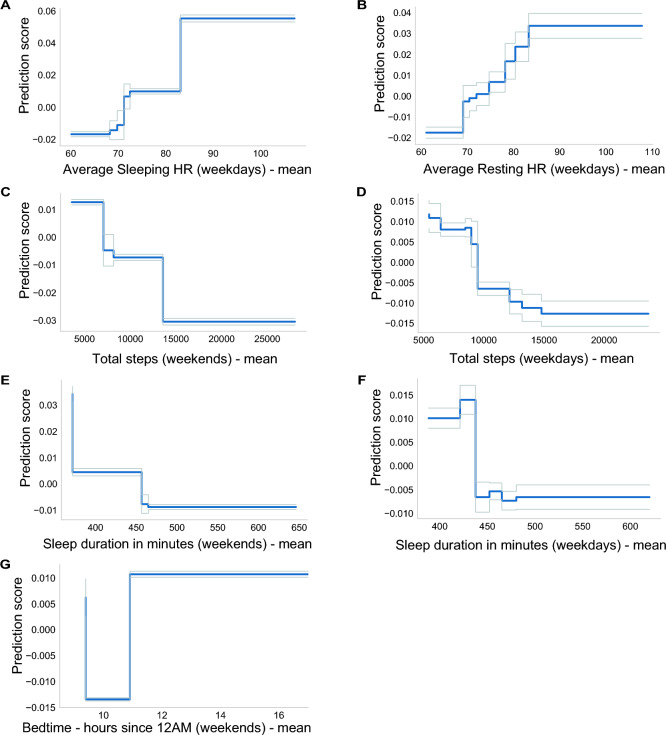
Contribution to the prediction (C) (blue line) for the predictors with the highest contribution in the best performing EBM: (**A**) Average Sleeping heart rate—weekdays (HR), (**B**) Average Resting heart rate—weekdays (HR). (**C**) Total step count—weekends, D. Total step count—weekdays, (**E**) Sleep duration in minutes—weekends, (**F**) Sleep duration in minutes—weekdays. (**G**) Bedtime in hours—weekends. The grey lines represent the standard error. These graphs showcase the nuanced effect of each variable on obesity prediction, highlighting zones where their influence shifts from being protective to risky, or vice versa, allowing us to identify tipping points.

Both White race (*C* = 0.014) and household income (*C* = 0.012) were identified as important predictors in our analysis. Having a high sleeping heart rate (*C* = 0.016) and a high daytime resting heart rate (*C* = 0.013) were also in the top 20 factors associated with obesity. Additionally, physical activity measures represented by lower weekend (C = 0.011) and weekday step count (C = 0.009) were also associated with obesity. Notably, adolescents with weekend step counts below 5,000 were found to have an elevated risk, and those who achieved at least 15,000 steps per day exhibited a relatively low risk for obesity (Fig. 5).

Sleep variables, comprising 8 out of the top-ranked variables, were strong predictors of obesity risk. Factors such as late bedtime (after 12 AM) (*C* = 0.012), higher social jetlag (*C*_*Relative Social Jetlag*_ = 0.010, *C*_*Absolute Social Jetlag*_ = 0.010), shorter sleep duration (< 6 h) on weekends (*C* = 0.009) and on weekdays (<7 hours, *C* = 0.008 ), higher variability in sleep onset on weekdays (*C* = 0.009), higher variability in mid-sleep timing (*C* = 0.010), and higher variability in sleep end on weekends (C = 0.008) were all associated with an elevated risk for obesity (Fig. S2). Conversely, longer sleep duration (> 7.5 h) emerged as a protective factor, underscoring the multifaceted relationship between sleep patterns and obesity (Fig. 5).

## Discussion

Utilizing a nonlinear approach, we identified crucial tipping points in the predictors where specific behaviors began to put adolescents at heightened risk for obesity in this demographically diverse sample of U.S. 10–14-year-olds. We found that obesity was relatively common (~ 14% in the Fitbit subgroup) and was accurately detected in a machine-learning model with wearable-based measures of short sleep duration, low step counts, and high heart rate together with sociodemographic indicators of disparities. Results indicate the value of using wearables over multiple days to determine associations of healthy behaviors like sleep and physical activity with obesity, and the relevance of sociodemographic factors in adolescents.

Our results are consistent with prior research that has shown racial/ethnic disparities in adolescent obesity risk, with Black and Hispanic/Latinx adolescents at higher risk^54–56^. In a nationally representative U.S. cohort, Rendall et al.^56^ investigated the distinct obesity trajectories between Hispanic and Black children and reported that one in four Hispanic and Black children was estimated to have obesity by 8th grade, and Hispanic and Black children with overweight and obesity are less likely to return to normal weight levels than are White children with overweight and obesity. In addition, consistent with prior research^57^, we found that low household income and difficulties with food access were associated with an increased risk of obesity, highlighting the persistent socioeconomic factors that may contribute to health disparities.

In our models, cardiovascular fitness measures were strongly associated with obesity, including higher resting and overnight heart rate and low day-to-day variability in heart rate. Daytime resting heart rate is commonly used as an indicator of long-term cardiovascular health and fitness^58^, and a lower heart rate during sleep also reflects robust cardiovascular health^59^. Elevated resting heart rate is associated with higher BMI^60^, waist circumference^58^, and body fat percentage in adolescents^61,62^. This relationship may be multifaceted and complex. On the one hand, a higher resting heart rate could be linked to lower physical activity levels^58,63^, potentially contributing to weight gain and obesity^64^. On the other hand, higher BMI itself might be associated with an elevated heart rate due to the extra workload on the heart^30^. It is essential to note, however, that our cross-sectional data do not permit a clear determination of causal directionality.

Longitudinal observational studies reveal a complex relationship between physical activity, BMI, and resting heart rate in adolescents. More active adolescents tend to have a lower prospective risk for obesity (see reference^65^ for a review). This is supported by our results on the physical activity measures. High physical activity levels measured on weekdays and weekends appeared to be protective (10,000 + daily average step count), and low activity was related to high obesity risk as measured by the step count, which has been previously reported as a reliable measure for energy expenditure in adults^66^. Furthermore, it was also reported that the resting heart rate and changes in the resting heart rate have prospective effects and may predispose individuals to the development of obesity both in adolescents^67^ and in adults^68^. In our analysis, both physical activity and resting heart rate were strong and independent predictors of obesity.

Later bedtime, short sleep duration, variability of mid-sleep timing, and variability in sleep onset and the endpoint of sleep (wake up) were identified as important measures associated with obesity. The association between short sleep duration and obesity in adolescence has been supported by cross-sectional^69–71^ and longitudinal studies^72^. Most of the previously reported results were based on self-reported data^69,70^ or utilized objective sleep measures with relatively low sample sizes^71^. This is the first study, to our knowledge, documenting the association between poor sleep health and obesity based on wearable sleep technology in a large sample of adolescents. Although there are multiple studies showing that sleep quality and timing are related to body fat percentage and obesity risk in youth^73^, awareness about the importance of sleep remains low in the general population^74^, leading to potential long-term consequences on their physical and mental health^75^. Our findings, which provide specific thresholds for bedtime and other health factors associated with obesity, can be particularly valuable for practitioners. These insights may inform both prevention and intervention strategies for obesity^76^, enhancing the ability to manage this complex health issue through a better understanding of the role of sleep.

The strengths of the present study include (i) the large and diverse adolescent sample and the length of the behavioral time series collected and analyzed, which is extensively higher than most previous studies analyzing obesity in adolescents, thus increasing the ecological validity of the present results; (ii) our modeling efforts, including the cross-validation and testing for non-linear associations; (iii) the choice to focus on step counts rather than energy equivalents, a decision informed by potential reliability issues with photoplethysmography, especially in individuals with high BMI^77^ or dark skin tone^78^.

The study also has limitations. First, because the Fitbit accurately detects sleep–wake states^36,59^, we focused on derived outcomes of sleep quantity and quality but did not investigate sleep architecture. These outcomes, however, cannot provide a full understanding of sleep behavior, and future studies are needed to get more insight into the sleep-related brain activities in obesity (i.e., polysomnography). Second, challenges in detecting sleep onset^79^ and potentially reduced reliability in individuals with sleep problems^80^ pose limitations on the Fitbit's ability to fully capture sleep patterns. Third, the lower representation of adolescents identifying as a race other than White who participated in the Fitbit sub-study^81^ indicates that our sample is not as diverse as the whole ABCD cohort, and there may be barriers to collecting these data in minorities. Lastly, the data are observational and cannot determine causality. Additionally, the inclusion of food intake and food logs could potentially increase the model performance; however, the current analysis did not include dietary information. Future work can extend this analysis with longitudinal data to determine whether sleep and physical activity measures predict the development of obesity over time, and thus offer more definitive insights.

## Conclusion

In conclusion, the present study demonstrated that objective sleep, physical activity, and cardiovascular measures collected by a commercial wearable device combined with sociodemographic factors are accurate predictors of early adolescent obesity. Our results showed that elevated resting heart rate, lower socioeconomic status (e.g., lower household income), minority race, shorter sleep duration and lower step count were associated with a higher risk for obesity. Measuring sleep, steps, and heart rate using wearable consumer devices is a viable alternative for large health monitoring systems because they are easier to employ than in-person clinic visits. Considering the prevalence of obesity and the associated sleep problems in adolescents in the general population, these devices could be incorporated into the clinical practice to increase awareness and support screening, diagnosis, and treatment in this age group to promote metabolic health, healthy sleep, and overall well-being, especially in the socially disadvantaged groups who may face barriers to in-person clinic visits. This could lead to earlier detection of obesity and associated health problems, as well as more effective treatment and management. Incorporating wearable devices into clinical practice may also promote healthy behaviors, such as increased physical activity and improved sleep, which were protective in our analysis and could have positive effects on overall health and well-being beyond obesity prevention.

### Supplementary Information

Supplementary Information.

## Data Availability

The datasets analyzed during the current study are from the ABCD data repository, available at the NIMH Data Archive Digital Object Identifier (DOI) 10.15154/8873-zj65. DOIs can be found at 10.15154/1518688.
