# Supplementary material for: Using explainable machine learning and fitbit data to investigate predictors of adolescent obesity
Source: Sci Rep. 2024 May 31;14:12563. doi: 10.1038/s41598-024-60811-2 (PMC11143310; doi:10.1038/s41598-024-60811-2)
Supplement: Supplementary file 1 — Supplementary Information. [file 41598_2024_60811_MOESM1_ESM.docx]

**Supplementary material**

**Title**: Using Explainable Machine Learning and Fitbit Data to Investigate Predictors of Adolescent Obesity

**Authors and affiliations**

Orsolya Kiss^1*^, Fiona C. Baker^1,5^, Robert Palovics^3^, Erin E. Dooley^4^, Kelley Pettee Gabriel^4^, Jason M. Nagata^2^

1. Center for Health Sciences, SRI International, Menlo Park, California, USA
2. Department of Pediatrics, University of California, San Francisco, San Francisco, California, USA
3. Department of Neurology and Neurological Sciences, Stanford University School of Medicine, Stanford, CA, USA
4. Department of Epidemiology, University of Alabama at Birmingham, 1665 University Boulevard, Birmingham, AL 35233, USA
5. School of Physiology, University of the Witwatersrand, Parktown, Johannesburg, South Africa


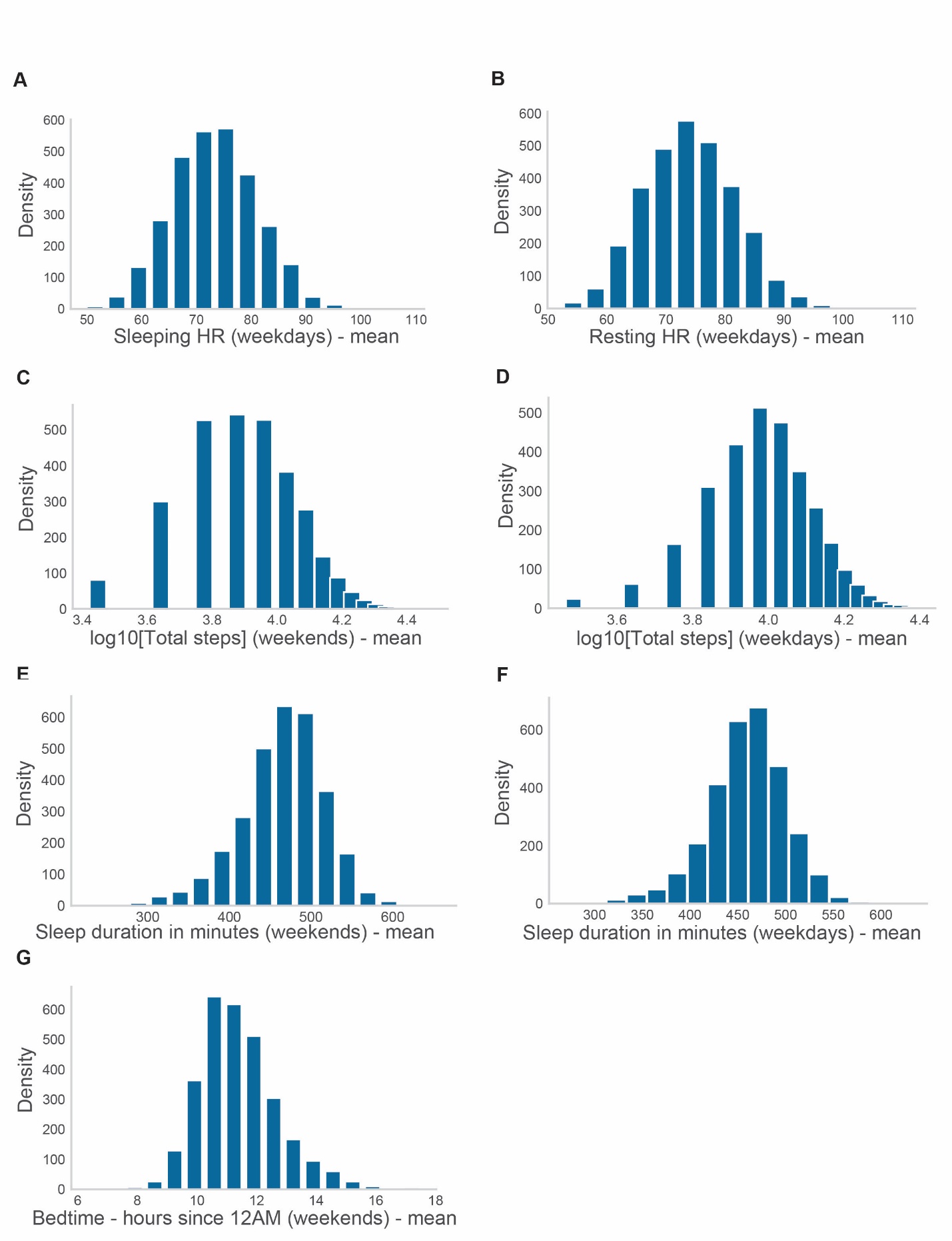


**Supplementary Figure 1.** Distribution of the predictors with the highest contribution scores in the best performing EBM: A. Sleeping heart rate - weekdays (HR). B: Resting heart rate - weekdays (HR), C. Total step count - weekends, D. Total step count - weekdays, E. Sleep duration in minutes - weekends F. Sleep duration in minutes – weekdays, G. Bedtime in hours - weekends.
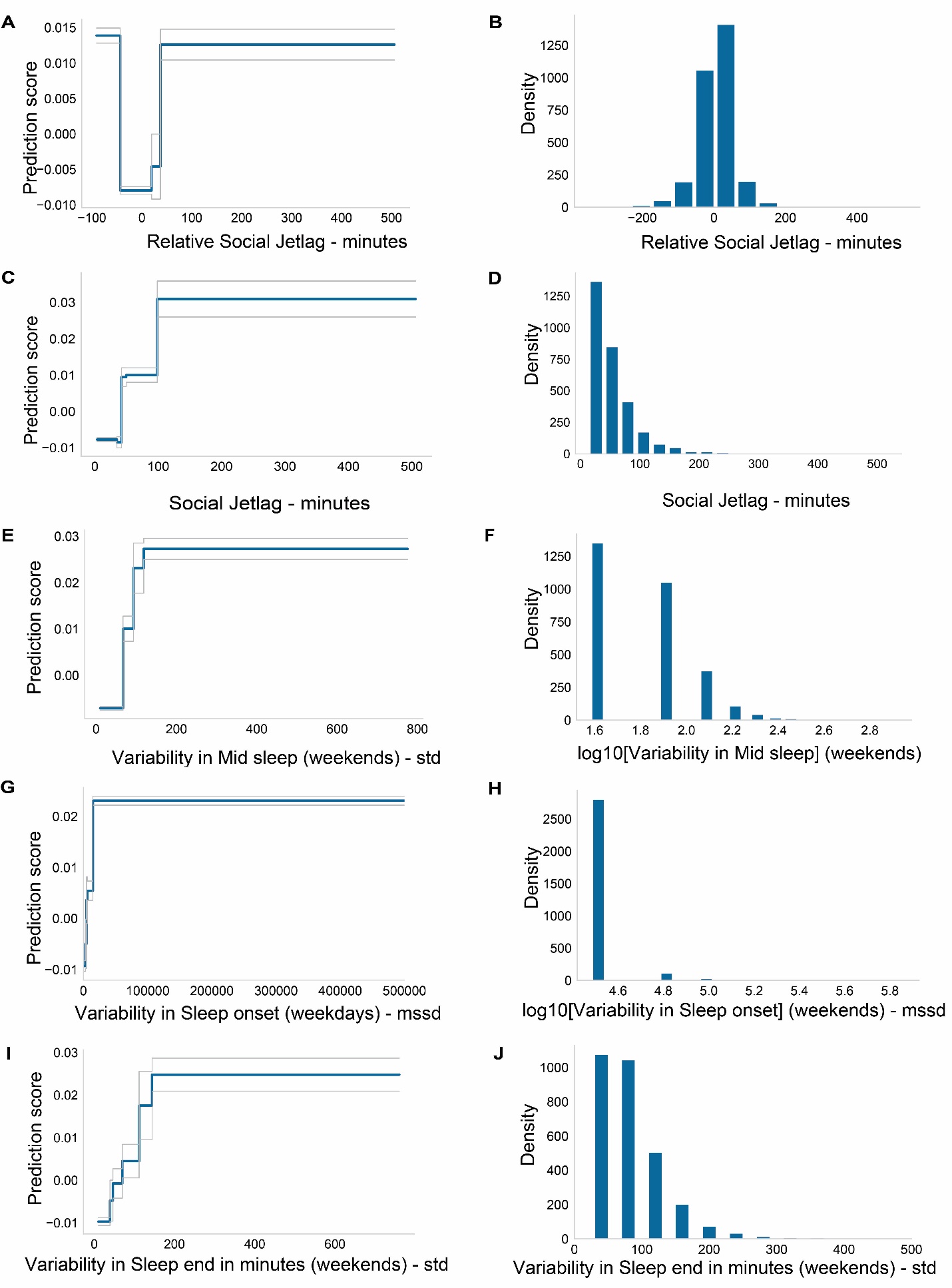


**Supplementary Figure 2.**  The contribution scores of the variability measures and their distributions in the best performing EBM: A. Relative social jetlag, B. Distribution of the Relative social jetlag, C. Absolute social jetlag, D. Distribution of the Absolute social jetlag, E. Variability in Mid sleep (STD), F. Distribution of the Mid sleep variability (STD), G. Variability in Sleep onset (MSSD), H. Distribution of the Sleep onset variability (MSSD), I. Variability in Sleep end (STD). J. Distribution of the Sleep end variability (STD). MSSD: mean square successive difference, STD: standard deviation.

**Section 1: Fitbit data collection and curation:** Our study utilized Fitbit devices to monitor physical activity and sleep patterns. The data collection spanned a three-week period, with both children and parents provided instructions on device maintenance. Data were synced to Fitabase in real-time and subsequently imported to ABCD servers securely. To ensure data integrity, research staff manually logged wear dates into both Fitabase and REDCap databases. Daily monitoring by research staff at each site via the Fitabase dashboard enabled the maintenance of continuous data collection and intervention when necessary to ensure device wear and data syncing. In instances of data inactivity exceeding three days, the research staff proactively engaged with participants to address any issues^1^.

**Preprocessing provided by the ABCD Data Management team**: We utilized the daily aggregated physical activity and sleep measures, as it was released by the ABCD Data Management team. Initial preprocessing and quality assurance were essential components handled by the ABCD data team to ensure the validity of the physical activity and sleep data collected from Fitbit devices. Detailed sleep stage measures were only provided by Fitbit if a sleep episode met a minimum threshold of 3 hours. Heart rate (HR) data were checked for missing values. For physical activity measures, any minute without HR data triggered an automatic generation of baseline values (0 for steps and 1 for sedentary in activity classification). Moreover, the dataset was screened for heart rate values falling outside physiological expectations (below 40 or above 200 beats per minute) to gauge the prevalence of unlikely physiological readings^1^. In line with NIH recommendations regarding handling accelerometer-based data, the identification of repeated heart rate values also formed a crucial part of the quality assurance process^1^. This involved recognizing sequences of identical HR readings or consistent HR values surrounding missing data, indicative of potential inaccuracies. Lastly, after a thorough validation, minutes that were part of repeated strings of 11+ minutes were excluded then daytime and nighttime wear were decoupled^1^.

**Section 2:** Our study participants were equipped with Fitbit devices for a continuous period of three weeks. An integral part of our data preprocessing involved a thorough examination of the alignment and consistency across the data collected during these weeks, as illustrated in Supplementary Figure 3. To ensure the integrity and reliability of our analysis, we opted to utilize the data from the second and third weeks. By focusing on these weeks, we believe that the data provide a more accurate representation of the participants' habitual sleep behaviors and physical activity levels, which are critical variables in predicting obesity.


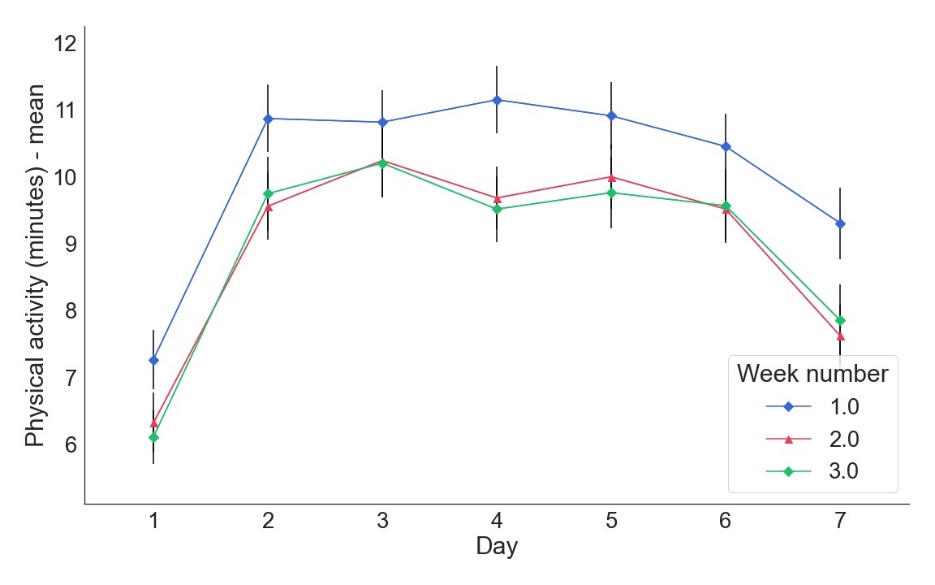


**Supplementary Figure 3**: Physical activity measures across the three weeks of data collection

**References:**

1. Wing, D.*, et al.* Recommendations for Identifying Valid Wear for Consumer-Level Wrist-Worn Activity Trackers and Acceptability of Extended Device Deployment in Children. *Sensors* **22**, 9189 (2022).
